# Supplementary material for: Wolbachia modifies thermal preference in Drosophila melanogaster
Source: Environ Microbiol. 2018 Oct 2;21(9):3259–68. doi: 10.1111/1462-2920.14347 (PMC6766989; doi:10.1111/1462-2920.14347)
Supplement: Supplementary file 2 — Fig. S1. Thermal gradient apparatus gradient depicting different temperature zones and fly dispersion (wMelPop). Fig. S2. Schematic of the thermal gradient apparatus used for thermal gradient assays as adapted from Rajpurohit and Schmidt (2016). The polycarbonate tube and length of aluminium gradient within the tube were 58.76 cm and temperature was recorded with K‐type thermocouples. Fig. S3. Average + 0.5°C (SD) temperatures from 18 runs that were recorded at each surface measured using k‐type thermocouples. There was a linear increase in temperature from cold to hot as measured at each of seven evenly spaced (8.39 cm). Fig. S4. Plots showing linearity of temperature change for the different surfaces (a. aluminium rod, b. top, c. bottom, and mid‐point of the polycarbonate tube) as measured with K‐type thermocouples at regular intervals along the length of apparatus from the hottest end (H3) to the coldest (C3). Fig. S5. Line plots showing the portion of flies observed at a given temperature for males and females in the left panel and age classes (young: 3–4 days or old: 10–14 days post eclosion) in the right panel. Each of the four subfigures shows the average proportion of flies with respect to different infection status (uninfected, wMel, wMelCS and wMelPop). For wMelPop infected flies only two age groups were tested. Error bars represent standard errors for average frequencies at a given temperature across all replicated experiments carried out for a given infection type and levels of the factors sex or age. Table S1. Counts of flies and number of replicates (in parentheses) per sex and age class. Table S2. Results of two‐way GLMM with independent factors age, Wolbachia and the interaction between them (see Table 2 for more detail). [file EMI-21-3259-s002.pdf]

SUPPORTING TABLES and FIGURES

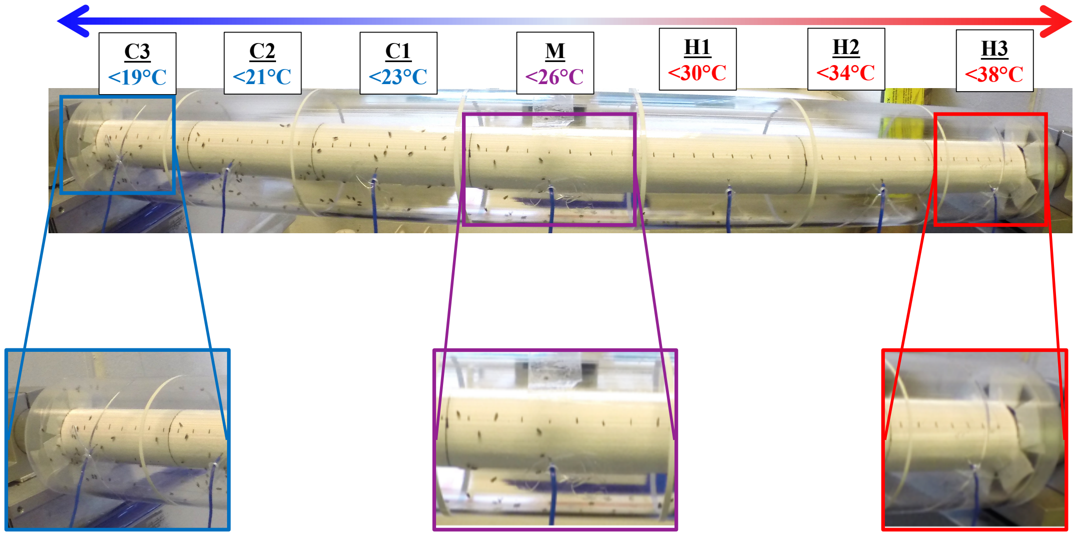

Figure S1: *Thermal* gradient apparatus gradient depicting different temperature zones and fly dispersion (*w*MelPop).

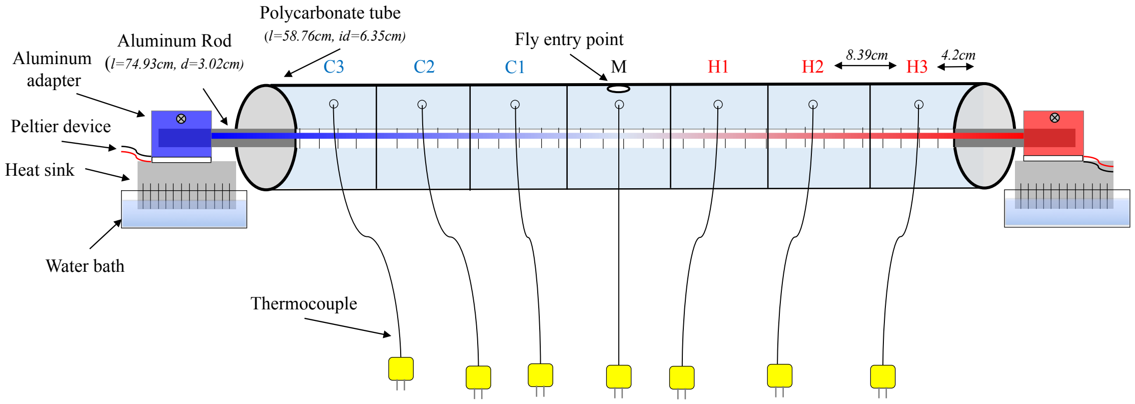

Figure S2: Schematic of the thermal gradient apparatus used for thermal gradient assays as adapted from Rajpurohit & Schmidt (2016). The polycarbonate tube and length of aluminum gradient within the tube were 58.76cm and temperature was recorded with K-type thermocouples.

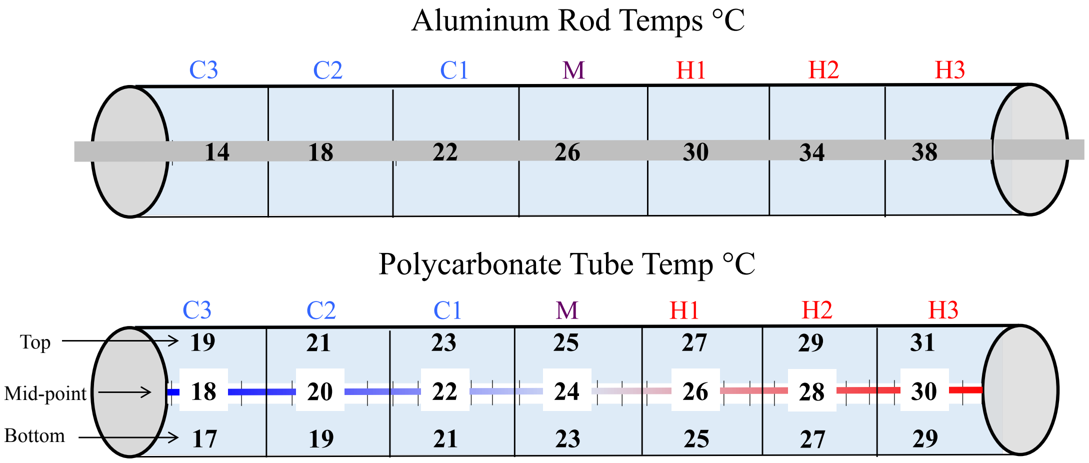

Figure S3: Average + 0.5°C (SD) temperatures from 18 runs that were recorded at each surface measured using k-type thermocouples. There was a linear increase in temperature from cold to hot as measured at each of seven evenly spaced (8.39cm).

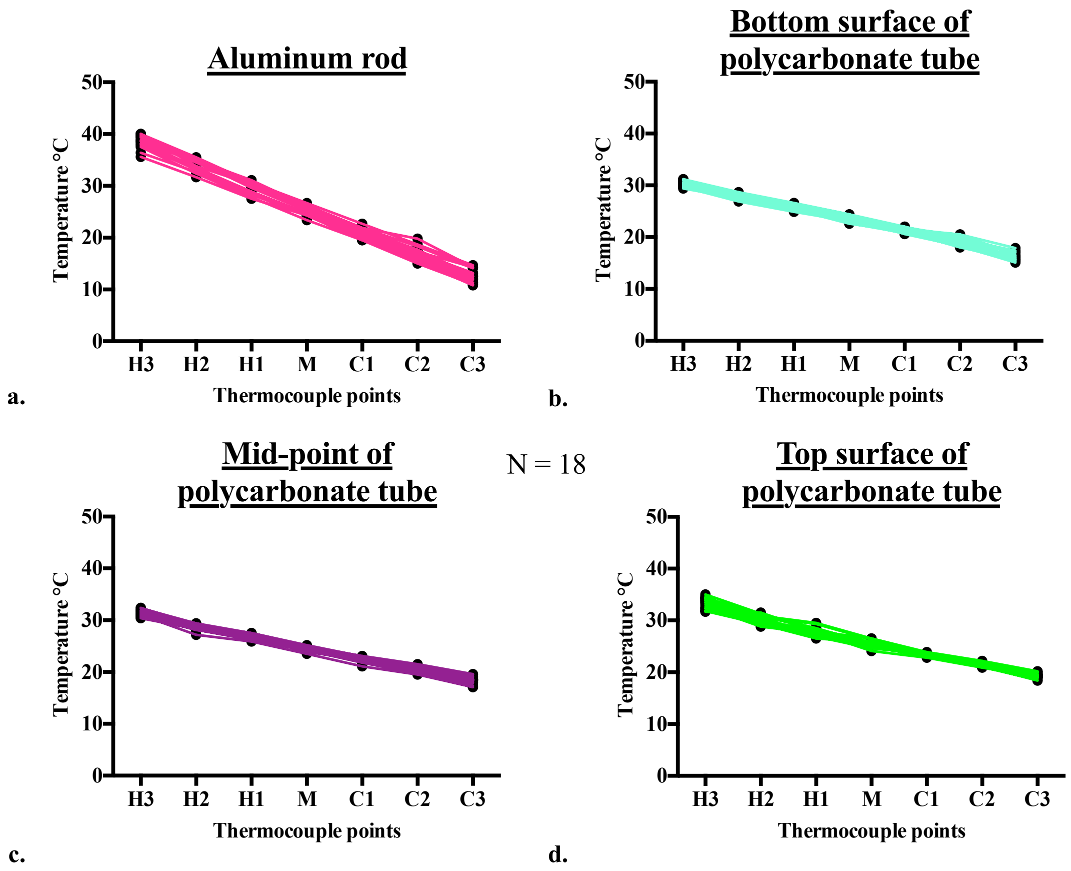

Figure S4: Plots showing linearity of temperature change for the different surfaces (a. aluminum rod, b. top, c. bottom, and mid-point of the polycarbonate tube) as measured with K-

type thermocouples at regular intervals along the length of apparatus from the hottest end (H3) to the coldest (C3).

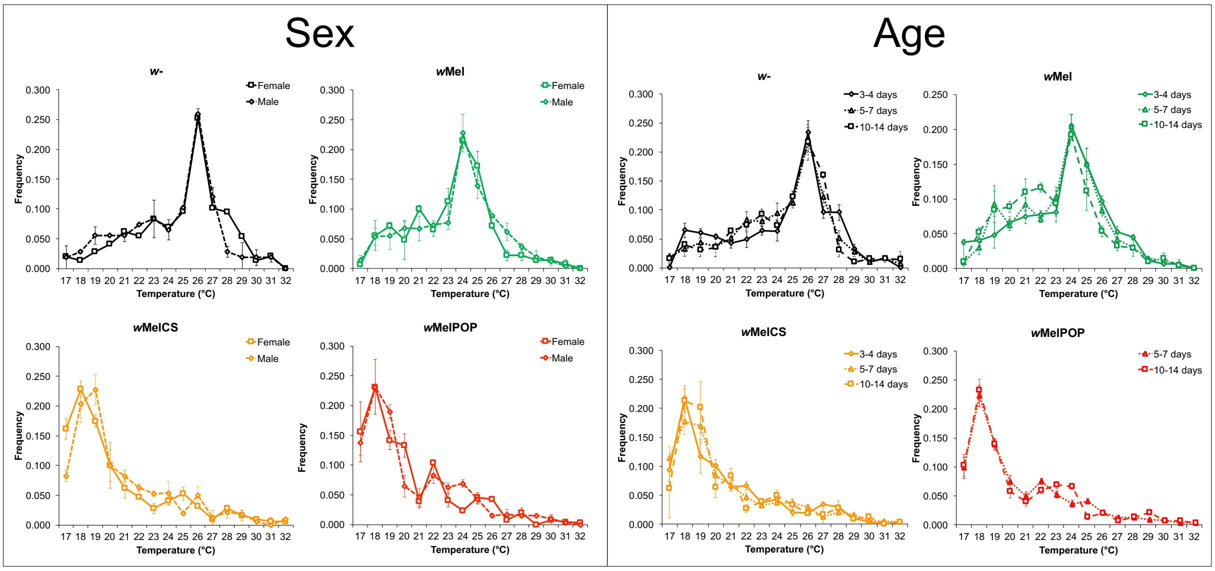

Figure S5: Line plots showing the portion of flies observed at a given temperature for males and females in the left panel and age classes (*young*: 3-4 days or *old*: 10-14 days post eclosion) in the right panel. Each of the four subfigures shows the average proportion of flies with respect to different infection status (uninfected, *w*Mel, *w*MelCS, and *w*MelPop). For *w*MelPop infected flies only two age groups were tested. Error bars represent standard errors for average frequencies at a given temperature across all replicated experiments carried out for a given infection type and levels of the factors sex or age.

***Table S1*:** Counts of flies and number of replicates (in parentheses) per sex and age class

**Counts**	**Females**	**Males**	**Unknown Sex**	**3-4 days**	**5-7 days**	**10-14 days**
***w*-**	147 (2)	108 (1)	1032 (10)	188 (2)	893 (9)	206 (2)
***w*Mel**	140 (2)	269 (3)	1055 (10)	211 (2)	986 (10)	267 (3)
***w*MelCS**	319 (3)	211 (2)	1089 (10)	276 (3)	1159 (10)	184 (3)
***w*MelPop**	260 (3)	264 (3)	823 (8)	0 (0)	1088 (11)	259 (3)

***Table S2*:** Results of two-way GLMM with independent factors age, *Wolbachia* and the interaction between them (see Table 2 for more detail)**.**

**Model**	**Factor**	**n**	**Df**	**$\chi 2$**	***P*-value**
wol* + *age* + *wol* x *age	***wol***	**5042**	**6**	**149.64**	**9.19E-30**
wol + *age* + *wol* x *age*	*age*	5042	4	0.39	0.98
wol + *age* + *wol* x *age*	*wol* x *age*	5042	3	0.17	0.98
